# Importance of GLUT Transporters in Disease Diagnosis and Treatment

**DOI:** 10.3390/ijms23158698

**Published:** 2022-08-04

**Authors:** Abdelrahman Ismail, Marina Tanasova

**Affiliations:** 1 Department of Chemistry, Michigan Technological University, 1400 Townsend Drive, Houghton, MI 49931, USA; 2 Health Research Institute, Michigan Technological University, Houghton, MI 49931, USA

**Keywords:** sugar transport, GLUTs, metabolic diseases, diagnostic biomarkers, GLUT inhibitors, GLUT therapeutics

## Abstract

Facilitative sugar transporters (GLUTs) are the primary method of sugar uptake in all mammalian cells. There are 14 different types of those transmembrane proteins, but they transport only a handful of substrates, mainly glucose and fructose. This overlap and redundancy contradict the natural tendency of cells to conserve energy and resources, and has led researchers to hypothesize that different GLUTs partake in more metabolic roles than just sugar transport into cells. Understanding those roles will lead to better therapeutics for a wide variety of diseases and disorders. In this review we highlight recent discoveries of the role GLUTs play in different diseases and disease treatments.

## 1. Overview of GLUTs

GLUTs are passive membrane transporters that pass glucose and other similar substrates like fructose, mannose, ascorbate, and urate ions. They have been classified into three classes based on their sequence similarity and substrate affinity. Table 1 [1] shows an overview of the 14 members of the GLUT family, their currently known substrates, and their expression sites. The Class I GLUTs (1–4 and 14) facilitate the uptake of glucose and other hexoses, but not fructose. The Class II GLUTs (5, 7, 9 and 11) are fructose transporters, and Class III GLUTs (6, 8, 10, 12, and 13 (HMIT1)) are structurally atypical members. The affinity of each transporter for glucose ranges from 0.2 to 17 mM.

Only GLUTs 1–5 have been studied in depth, and relatively little is known about the other GLUTs. This section provides an overview of the transport efficiencies, tissue expression, and links to different disorders of all 14 GLUTs.

### 1.1. Class I

GLUT1 was the first GLUT to be identified. It is ubiquitously found in all tissues of the body. GLUT1 also transports glucose through the blood-brain barrier [2,3], and is expressed in other barrier structures in the brain [4]. Its main substrate is glucose (*K*_m_ = 3 mM [5]), and other known substrates include galactose, mannose, and glucosamine [6]. GLUT1 is overexpressed in many different types of cancers including brain [7], breast [8], cervix [9], colon [10], kidney [11], lung [12], ovary [13], prostate [14], skin [15], and thyroid [16]. Cancers that express more GLUT1 have been shown to be more aggressive and invasive [17]. Due to its widespread prevalence, many different therapies have attempted to target it, but the same widespread prevalence makes it difficult to achieve specificity.

GLUT2 has a sequence similarity of 55% to GLUT1. It is primarily expressed in the liver, kidney, insulin-secreting pancreatic beta cells, and absorptive epithelial cells of the intestinal mucosa. Its main function is regulating the uptake of glucose in the gastrointestinal tract [18,19]. GLUT2 partakes in glucose uptake (*K*_m_ = 17 mM), but its main substrate is glucosamine (*K*_m_ = 0.8 mM) [6]. It also transports fructose, galactose, and mannose (*K*_m_ = 76 mM, 92 mM and 125 mM, respectively) [20,21,22]. Since it plays a significant role in carbohydrate uptake in the intestines, it has become a target of interest for diabetes prevention and treatment by inhibiting glucose absorption in the intestine and thereby lowering blood glucose levels [23].

GLUT3 is the second most prevalent transporter in the brain, but unlike GLUT1, it is widely distributed in the neurons [24,25], particularly in the pre- and post-synaptic nerve terminals and small neuronal processes [26]. It is also expressed in embryos, sperm, and white blood cells [27]. It has the highest affinity to glucose of all class I transporters (*K*_m_ = 1.4 mM) [28]. It was also shown to transport mannose, galactose, and xylose [28]. GLUT3 is overexpressed in many cancers including breast, colon, endometrial, kidney, lung, and renal cancers [8,29,30]. This overexpression is also associated with the aggressiveness of glioblastomas and recurrent brain tumors [31].

GLUT4 is the dominant glucose transporter in striated muscle and adipose tissues, and is the second most abundant transporter in cardiovascular tissue [32]. Its main substrate is glucose (*K*_m_ ≈ 5 mM [33]) and it also transports mannose, galactose, dehydroascorbic acid, and glucosamine [34]. Unlike other glucose transporters, GLUT4 is regulated by insulin as insulin binding receptors translocate GLUT4 to the cell surface [27]. GLUT4 has been linked to obesity, type-2 diabetes, and heart disease, making inhibition of GLUT4 a promising therapeutic approach [35,36]. In fact, GLUT4 inhibition has been shown to cause cardioprotective effects and aided affected individuals to return to normal heart/body weight ratios [35].

GLUT14 is the most recently identified member of the GLUT family. It is a duplicon of GLUT3 but it is only expressed in the testis [37]. Not much else is known about this transporter.

In addition to the expression regulation by their substrates, the expression of GLUTs 1–4 was also found to be regulated by hormones. Estrogen and progesterone have been linked to GLUT expression in the endometrium [38]. Data suggests that high glucose uptake and metabolism are necessary for endometrial proliferation and differentiation. Abnormal GLUT expression has been found in a wide range of endometrial cancers, and steroid hormones have been linked to the genesis of endometrial cancer [39].

### 1.2. Class II

While fructose is a rare substrate for the class I GLUTs, it is one of the main substrates of class II GLUTs. Class II GLUTs also have significantly higher affinities for their substrates in general, including glucose. Among all GLUTs, GLUT5 is a unique transporter, as its only substrate is fructose (K*i* = 5–15 mM [40]). It is primarily expressed in the small intestine [41] and has been strongly linked to cancer development, progression and metastasis, making it an attractive target for cancer therapeutics [42]. This, along with its unique substrate specificity, prompted several structural activity relationship (SAR) studies into its H-bonding requirements for fructose uptake in order to design GLUT5-specific probes as cancer diagnostic tools [43,44,45]. Furthermore, the heightened consumption of fructose in cancers lead to the development of GLUT5-specific inhibitors of fructose uptake [42,46].

GLUT7 is primarily expressed in the small intestine and colon, although its mRNA has been detected in the prostate and testis. It has a sequence identity of 44% to GLUT5 [47]. GLUTs 9 and 11 share 58.1% and 41.7% sequence identity with GLUT5, respectively [40,48]. All three transporters have high affinities (<0.5 mM) for both glucose and fructose [47,49]. GLUT9 is mainly expressed in the liver and kidney [49], and GLUT11 has been found in various organs, including the heart, skeletal muscle, kidney and pancreas [50]. GLUTs 7 and 9 are also expressed in the apical membrane of the small intestine and colon. The abundance of GLUTs 7 and 9 in the small intestine changes according to dietary carbohydrate intake. However, the distribution of transporters along the small intestine does not entirely match the availability of glucose and fructose, which might indicate the presence of another substrate for those transporters that has yet to be identified.

### 1.3. Class III

The members of this class share a limited sequence homology with class I (~25% identity) and are considered structurally atypical [51]. GLUT10 has a 35% similarity to GLUT2 and it can also transport glucose (*K*_m_ = 2 mM). GLUT12 has a number of similar features to GLUT4, but a much higher affinity for glucose (*K*_m_ = 0.3 mM) [52].

GLUT8 is predominantly expressed in the testes [53] and is thought to play a major role in providing glucose to mature spermatozoa, in addition to being able to transport fructose [54]. It is also expressed in a number of other tissues such as the liver, spleen, brown adipose and blastocysts, albeit in significantly lower quantities [55]. The expression of GLUT8 was suggested to be regulated by insulin in a similar manner to GLUT4. However, this claim has been disputed [56,57]. There is some evidence that glucose itself may influence the location and expression of GLUT8, as glucose induces GLUT8 translocation from an intracellular compartment to the endocyclic reticulum in rat hippocampal cells [58]. GLUT8 knock-out mice reportedly displayed normal embryonic and postnatal development and glucose homeostasis, but had mild defects in hippocampal neurogenesis and cardiac function.

GLUT12, like GLUT8, is also able to transport both glucose and fructose. Insulin-regulated expression has also been suggested for GLUT12, as it is predominantly expressed in the insulin-sensitive skeletal muscle, heart and fat tissues [52]. GLUT12 was originally cloned from the human breast cancer cell line, MCF7, and its expression was found to be stronger in ductal cell carcinoma than in benign ducts of breast cancer tissues [59], indicating a possible role in glucose uptake in breast cancer tissue.

HMIT, formerly known as GLUT13, is an H^+^/myo-inositol cotransporter and is the last of the GLUTs. Unlike the other 13 transporters, HMIT does not transport either glucose or fructose. Its only substrate is myo-inositol (*K*_m_ ≈ 100 mM), and this uptake is pH-dependent [60]. HMIT is largely expressed in the brain, particularly in neuron intracellular vesicles, and its transition to the cell surface can be triggered by cell depolarization.

## 2. Carbohydrate Uptake and Metabolic Disorders

Increased carbohydrate consumption is implicated in a variety of diseases, including obesity, type 2 diabetes [61], nonalcoholic fatty liver disease [62], gout, sclerosis, and Alzheimer’s disease [63]. High glucose or fructose uptake are associated with increased cancer growth and metastasis [64,65]. Therefore, the changes in GLUT expression and composition occur to meet the additional energy demands of uncontrolled cell growth and division. For example, the expression of the normally ubiquitous GLUT1becomes unpredictable, with some cancers overexpressing this transporter and others downregulating it [66]. Thus, GLUT1 has been found to be overexpressed in the squamous cell carcinoma of lung cancer, but not in normal or adenocarcinoma lung cells [67]. Likewise, GLUT3 gains expression in various types of cancer, while normally being only expressed in the brain [68]. The overexpression of GLUT5 and GLUT12 bas been observed in both the early-stage breast carcinoma cell line MCF7, and the late-stage cell line MDA-MB-231 [69,70]. GLUT5 is similarly overexpressed in pancreatic, ovarian, and lung cancers and leukemia [42,71].

Despite this, the relationship between sugar consumption and the development of cancer is not clear cut. While it was shown that a diet high in fats and sugars contributes to the reoccurrence of stage III colon cancer, it did not have any impact on the overall risk of colon or any other type of cancer, or it showed an increased risk in only a narrow population. On the other hand, high sugar consumption is strongly linked to obesity, and there is an established link between obesity and cancer [72]. Increased circulating insulin and insulin-like growth factor levels also have been linked with cancer progression, suggesting that both obesity and insulin resistance might promote cancer development by activating cell growth signaling pathways [73,74].

Alterations in sugar uptake in metabolically-compromised cells led to the development of sugar transport-targeting therapeutic approaches. Cell starvation in sugars was found to diminish the viability of cancer cells [75] and improve therapeutic outcomes [76]. The approach relied on the resilience of normal cells to the nutrient deprivation, while cancer cells remained vulnerable [77]. The induced stress in cancer cells was observed to play a role of a sensitizer and enhance their response to chemotherapy agents [77]. Such a cancer sensitization strategy promoted the development of ketogenic diets that allow for the limiting of sugar consumption [78]. Along with the development of dietary approaches, direct methods to modulate sugar uptake in cells have been developed. This approach relies on a direct inhibition of sugar uptake in cells using GLUT inhibitors. Several comprehensive reviews have summarized the natural or synthetic GLUT inhibitors [1,79,80]. Downregulating the expression of selected GLUTs has also been proposed as a potential therapeutic strategy. Over the last several years, significant advances have been made towards diversifying the library of inhibitors in addition to streamlining their specificity as well as broadening the assessment of GLUT inhibition outcomes. In addition, new connections between GLUTs and disease have recently been identified, which could lead to new additional diagnostic and therapeutic approaches. An update of recent advances for several different diseases (in alphabetical order) follows below.

## 3. Recent Examples of GLUT Associated Diseases

### 3.1. Arterial Tortuosity Syndrome

ATS is a connective tissue disorder that causes tortuosity and aneurysm formation in the major arteries. It is a rare genetic disorder caused by mutations in the GLUT10 gene that cause it to stop functioning. Researchers in the past failed to induce ATS in mice using GLUT10 knockout, since mice are able to synthesize dehydroascorbic acid, one of the substrates of GLUT10. By contrast, humans cannot synthesize dehydroascorbic acid, so the authors did a double knockout of GLUT10 and L-gulonolactone oxidase, a critical enzyme for ascorbic acid synthesis in mice. The vasculature of double knockout, single knockout, and wild type mice was measured by ultrasound. For double knockout mice of both genders of mice across all measured blood vessels, the diameter of the vessels was 1 mm smaller on average. Those deviations were not enough to be considered as a reproduction of the major hallmarks of ATS in humans. However, an in vitro culture showed that DKO mice had weakened extracellular matrix structures, with fewer and thinner collagen links compared to the WT mice.

GLUT10 knockout has been previously associated with impaired mitochondrial function, and this model does demonstrate that when comparing the oxygen consumption rate of the DKO mice and WT mice, the basal consumption of DKO mice was about 70 pmol/min lower than the WT, while the maximum rate was lower by as much as 350 pmol/min, a difference of more than three-fold [81]. This work demonstrated the importance of GLUT10 in dehydroascorbic acid transport and mitochondrial function; the underlying mechanism of the latter remains unknown. Further research is needed to develop a better model for ATS as well as to understand the link between GLUT10 and mitochondrial function.

### 3.2. Cancer

#### 3.2.1. Breast Cancer

Triple-negative breast cancer (TNBC) is infamous for its aggression, poor prognosis, and limited treatment options. How much TNBC relies on glycolysis had been largely unknown until recently, when it was shown that GLUT12 plays a key role in TNBC growth and was associated with worse clinical outcomes for TNBC patients. Analyzing 193 samples of TNBC tumors and 864 non-TNBC tumors, the former had an average of 27.5% higher GLUT12 expression (*p* = 2.7 × 10^−36^). In addition, a sample of 78 TNBC patients with high GLUT12 expression were able to survive for up to 100 months, which is half as long as 107 patients with low GLUT12 expression (*p* = 0.041). This data highlighted the need for TNBC therapies that target GLUT12.

A microRNA (miRNA) sequence called let-7a-5p was identified in silico to suppress GLUT12 expression. This was confirmed in-vitro using HEK293T cells, which when given let-7a-5p showed only one third of the GLUT12 expression of the negative control. Next, it was found that let-7a-5p reduced the proliferation of MDA-MB-231 and MDA-MB-468 cells by half after four days compared to the negative control. Restoring GLUT12 expression resulted in near identical proliferation to the negative control. Finally, nude mice (*n* = 6) with grafted MDA-MB-231 cells expressing firefly luciferase were imaged at 30 days. The mice that received let-7a-5p had visibly lower florescence compared to mice given scrambled miRNA. Dissecting their lungs showed they had an average of 10 tumor nodules compared to 40 for the control [82]. This study demonstrates the importance of GLUT12 and the role it plays in TNBC, and how it offers a new promising therapeutic pathway.

Another study looked at RIP140, a protein that, among many other functions, suppresses cell division and cellular metabolism, and cancer cells are often deficient in that protein. Transformed MEF cells with RIP140 knockout had a tumor volume of 2500 mm^3^ on average after 40 days in mice, while the wild type volume was only 500 mm^3^, a difference of fivefold. The tumor weights were 1.3 g and 0.4 g, respectively.

To investigate the mechanism behind the action of RIP-140, the expression of 23 different metabolites involved in cellular respiration were monitored using RT-qPCR in wild type and RIPKO immortalized MEF cells. GLUT3 expression was found to have increased more than two-fold in RIPKO compared to the wild type control. Silencing RIP-140 expression resulted in doubling the GLUT3 expression in the breast cancer cell lines MCF7 and MDA-MB-436. Moreover, analyzing the RNAseq data obtained from 1068 breast tumor samples on the TCGA dataset showed that higher RIP140 expression resulted in 20% higher probability to survive for 60 months. Having demonstrated the correlation between RIP140 levels and GLUT3 expression, the authors proposed that GLUT3 can be used as a prognostic tool that can help oncologists identify patients with low RIP140 levels who would benefit the most from treatments that target glycolysis [83].

#### 3.2.2. Colon Cancer

With no effective therapeutics in place, colon cancer is one of the leading causes of death in cancer patients worldwide. One study found that GLUT5 mRNA levels in colon cancer specimens from 30 different patients were double those of normal colon epithelial cells. It was also found that treating the cancer cell line HT-29 with the GLUT5 inhibitor MSNBA caused a 50% drop in cell viability at just 1 µM of inhibitor. Higher concentrations showed diminishing returns and also started to affect the viability of normal cells [84].

A more recent study helped uncover the role that GLUT5 plays in colon cancer proliferation and resistance to chemotherapy. GLUT5 is a fructose only transporter, and uptake studies with HCT116 and HT29 cancer cell lines showed that when glucose is absent, both of them exhibited nearly equal growth and proliferation compared to when glucose is present, as long as fructose is also present. This means that the cells were able to use fructose exclusively as their only energy source. Meanwhile, the absence of fructose reduced the proliferation of both cell lines by only 7% on average.

**Figure 1:**
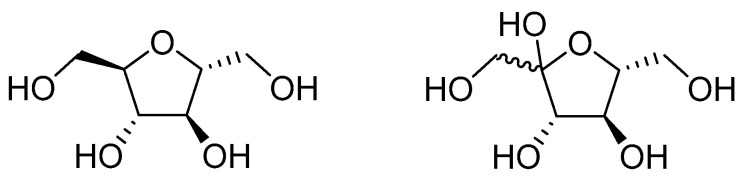
The structure of 2,5-anhydro-d-mannitol (**left**) compared to the structure of fructose.

To further investigate GLUT5′s role in the tumor’s growth and proliferation, the expression of enzymes involved in fructose metabolism was measured. It was found that GLUT5 increases the expression of ketohexokinase and prevents its degradation in the cells. This increase is dependent on the concentration of fructose in the culture medium, which in turn affects the expression of GLUT5. Silencing ketohexokinase significantly lowered the growth and proliferation of Caco2 and SW480 cells in 10 mM fructose and 1 mM glucose media, but this was not observed in 10 mM fructose and 10 mM glucose media. Finally, treating CRC cells with the fructose analog 2, 5-anhydro-*d*-mannitol (Figure 1) lead to a significant reduction in growth and proliferation by about 88% at 6 mM and 60% at 2 mM. This treatment also greatly improved the effectiveness of chemotherapeutic agents like cis-platin and oxaliplatin, reducing cell proliferation by 75% compared to having each chemotherapeutic drug on its own [85]. These studies highlight the crucial role GLUT5 plays in colon cancer and the potential of GLUT5 targeting therapeutics to improve clinical outcomes for patients by using them as chemotherapy adjuvants.

#### 3.2.3. Endometrial Cancer

GLUT6 was found to be overexpressed in endometrial cancers by about 37-fold compared to healthy tissue, and GLUT6 knockdown resulted in cell death, an indication that GLUT6 plays a critical role in the growth and metastasis of this cancer and would be an attractive therapeutic target. To identify the regulators of GLUT6 expression, the authors used a SureFIND transcriptome PCR array which contains cDNA from MCF7 cells. The impact of knocking down 270 transcription factors on GLUT6 expression was measured and it was found that RELA caused the greatest decline in GLUT6 expression by nearly 30-fold. Next, RELA was expressed in the normal endometrial epithelial cell line hUE-T, and a 35% increase in GLUT6 expression was observed, meaning that RELA alone can promote GLUT6 expression. TNFα, a pleotropic inflammatory molecule that is abundant in endometrial cancer patients, was also tested on hUE-T cells and it was found that only a dose of 2.5 ng/mL caused the biggest spike in GLUT6 expression by nearly 20-fold, and a bigger dose like 20 ng/mL only caused a nine-fold increase. In addition, TNFα treatment caused the phosphorylation of RELA at Ser536, which suggests the activation of the NF-κB signaling pathway. The authors concluded that the NF-κB signaling pathway plays an important role in GLUT6 expression, providing further rationale to investigate and develop therapeutics that target GLUT6 to treat this type of cancer [86].

#### 3.2.4. Gastric Cancer

Several natural product extracts are known to inhibit the growth of this cancer, like GRg3, which is found in ginseng, but their mechanism of action remains unknown. GRg3 was shown in previous studies to have antioxidant and anti-inflammatory properties, as well as inhibiting tumor metastasis and increasing the expression of pro-apoptotic factors, making it an attractive candidate to study further. The effect of GRg3 on GLUT expression in normal gastric mucosa cells and gastric precancerous lesion cells were measured. This experiment was done for GLUTs 1, 3, 4, 6, 10, and 12. Only GLUT1 and GLUT4 were found to be overexpressed in the malignant cells compared to the healthy cells by 2.4-fold and 6-fold, respectively. Malignant cells treated with GRg3 had very close GLUT1 expression to the healthy cells, while GLUT4 expression remained higher than the healthy cells at nearly two-fold, although it was four-fold lower than malignant cells with no treatment. To further corroborate this evidence, the cancer cell lines AGS and HGC-27 were treated with GRg3 and their GLUT expression was measured. The GLUT1 expression of AGS was half of the negative control, and GLUT4 expression was lower by about 40%. HGC-27 cells had nearly identical results, with GLUT1 expression being halved and GLUT4 expression being 40% lower. The expression of the other 4 GLUTs that were measured remained largely unchanged. The authors concluded that GRg3 is not only a potentially effective therapeutic for gastric cancer, but might also halt and reverse the development of precancerous lesions, which could help reduce the incidence of this type of cancer [87].

Along a similar vein, the combination of *Coptis chinensis* (CC) and dried ginger (DG) is used in traditional Chinese medicine to treat colds, gastric ulcers, and colitis. To understand the mechanism behind the properties of CC-DG, the human gastric cell line SGC7901 was treated with several CC-DG extracts in different proportions, and it was found that a 1:1 CC-DG ratio provided the maximum inhibition. Treatments with more CC came very close to the results of the 1:1 ratio, with 24:1 having 90% of the inhibitory effect of the 1:1 treatment. Treatments with more DG, on the other hand, lost their effectiveness very rapidly, with the 1:3 ratio having just 60% of the 1:1 inhibitory effect. Next, a cell viability assay using this 1:1 CC-DG ratio was carried out on SGC7901 cells and also the mouse gastric carcinoma cell line MFC. Both cell lines exhibited a significant reduction in viability after 24 h in a linear dose-dependent manner. At the maximum dose of 2000 μg/mL, both cell lines had a viability of about 40% and 37%, respectively. Following this, the impact of CC-DG on cellular metabolites was tested, and it was found that it significantly reduces GLUT1 and LDHA expression by 55% and 50% respectively in SGC7901 cells. Finally, MFC cells were transplanted in mice and given different doses of the CC-DG treatment over 22 days. The mice given the 50 mg/kg dose had nearly 40% lower tumor volume and half the tumor weight, while the mice given 100 mg/kg dose had 66% lower tumor volume and 87% lower weight. The authors concluded that the CC-DG medicine has potent anti-tumor effects that target glucose metabolism and thus may prevent or suppress gastric cancer [88].

#### 3.2.5. Leukemia

H22954, a novel non-coding RNA, was previously shown by the authors to inhibit the growth and induce apoptosis in leukemia cells. To further understand the underlying mechanisms, an F-FDG uptake assay was carried out on K562 cells with or without H22954. It was found that the treated cells had a 5% lower uptake than the untreated control. To identify which transporter was affected by H22954, the gene expression profile of stable K562 cells were compared with and without overexpressed H22954. Only GLUT10 expression was found to have decreased more than two-fold, while the rest of the GLUT transporters were mostly unaffected. Next, the cells were given actinomycin D to prevent the synthesis of new RNA, then the levels of GLUT10 were measured with and without H22954 treatment. After 30 min, less than 50% of the initial GLUT10 RNA remained. This suggests that H22954 reduces GLUT10 expression by accelerating the degradation of its RNA. Finally, blocking the H22954 and GLUT10 RNA interaction sites resulted in fully restored GLUT10 levels. In conclusion, H22954 has been shown to be a GLUT10 specific target that could help develop future leukemia treatments [89].

#### 3.2.6. Multiple Cancer GLUT Inhibitor

**Figure 2:**
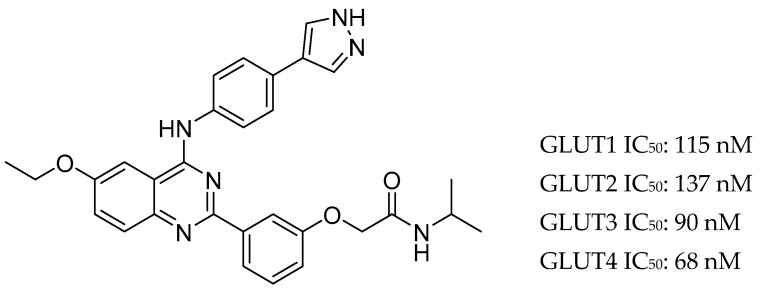
The structure of KL-11743 and its IC_50_ values against all class I GLUTs.

KL-11743 is a newly identified broad spectrum class I GLUT inhibitor (Figure 2). It is orally bioavailable and well tolerated for doses up to 100 mg/kg/day with no death or adverse effects. Treating the fibrosarcoma cell line HT-1080 with this inhibitor showed a potent dose-dependent growth inhibition (IC_50_ = 677 nM). Moreover, the cellular consumption of glutamine increased with the dose of inhibitor, going up by 40% at 3 μM of inhibitor. This suggests that the cells are using glutamine as an alternative fuel source. 

Testing different cell lines revealed that while HT-1080 cells require co-treatment with oligomycin to block ATP production, the renal cancer cell lines UOK-262 and UOK-269 undergo acute loss of ATP when treated with KL-11743, which drops to nearly zero at just 10 μM of inhibitor after 1 h. The inhibitor was also found to be cytotoxic for both these cell lines with LD_50_ values of 1130 and 565 nM respectively. Those effects were attributed to the impaired TCA cycle that both cell lines have as a result of homozygous null mutations in FH or SDH genes. As the authors were unable to graft HT-1080 in mice successfully, their TCA cycle deficient tumor lines were identified and grafted then treated with 100 mg/kg of KL-11743. The head and neck tumor HN0586 was about 22% smaller than the control after 42 days, the melanoma tumor ME12217 was 57% smaller after 20 days, and the lung tumor LU6415 was 48% smaller after 15 days. The authors concluded that GLUT inhibitors for cancer treatments would exhibit the best outcomes in TCA cycle deficient tumors [90].

### 3.3. Cognitive Decline

One of the risk factors for developing cognitive decline is Apolipoprotein E E4 (APOE4), and a high blood ascorbic acid level correlates with a reduction of APOE4 associated cognitive decline. Ascorbic acid is transported into the brain by the SVCT transporter, while the oxidized form dehydroascorbic acid is transported by GLUTs, mainly GLUTs 1 to 3 in the brain. In this research, 730 Japanese participants aged 65 or older were recruited in the study between 2006 and 2008. Their blood and DNA samples were taken and they were all confirmed to have normal cognitive abilities. The subjects were divided into three genotypes based on APOE4 expression and four groups based on GLUT and SVCT levels, which were determined from gene banks. Then, between 2014 and 2016, a follow-up cognitive exam with 400 participants revealed that 252 of them retained their normal cognition, 141 were showing symptoms of MCI or dementia, and seven subjects had inconclusive results. The researchers found the highest risk of APOE4 cognitive decline in the groups with low SVCT expression. By contrast, there was a higher risk in the groups with higher GLUT expression (Table 2) despite its involvement in ascorbic acid metabolism. However, the authors note that the correlation between GLUT levels and dehydroascorbic acid levels in the brain have not yet been established, given how GLUTs are nonspecific transporters and have other substrates, mainly sugars. Finally, the authors called for the investigation of the relationship between cognitive decline, dehydroascorbic acid, and GLUT expression in animal models [91].

### 3.4. Chron’s Disease

The increased presence of fructose in modern Western diets is suspected to have played a role in the increasing prevalence of inflammatory bowel diseases. A recent study provided evidence to support that hypothesis by feeding a 15 kcal% fructose diet to two groups of mice with different GLUT5 expression levels. It was found that the mice with lower GLUT5 levels experienced worse experimental colitis. Subsequent treatment of those mice with broad spectrum antibiotics protected against worsening symptoms. Those findings indicate that lower GLUT5 expression and dietary fructose intake both increase the risk of experimental colitis by affecting the gut microbiota composition [92]. 

### 3.5. Epilepsy

More than 50 million people worldwide suffer from epilepsy, and 50% of all epilepsies in humans were found to have a genetic underlying cause. Most commonly, mutations in ion channels including sodium, potassium, calcium, and chloride channels were associated with epilepsy, with the potassium channel in particular being the most commonly associated type. On the other hand, mutations in GLUT1 that lower its function were found in 12% of patients with early-onset absence epilepsy [93]. GLUT1 deficiency syndrome has also been linked with epilepsy, along with other disorders like microcephaly and developmental delay. A ketogenic diet has been found to be an effective treatment for GLUT1 deficiency and was recommended as a first line treatment for this disorder [94].

### 3.6. Hepatic Steatosis

Nearly a quarter of the world population suffers from non-alcoholic fatty liver disease, and its increased incidence is being blamed on the higher consumption of fructose in modern western diets. TM4SF5 is a is a membrane glycoprotein that is known to be involved in several forms of steatosis resulting from a high-fat diet or a methionine-choline deficient diet. However, its role in steatosis involving a high fructose diet is unknown. It was previously shown that TM4SF5 knockout mice on a high sucrose diet had lower hepatic accumulation of triacylglycerols compared to wild type mice on the same diet. It was also found that GLUT8 changes its intracellular location depending on the presence of TM4SF5. To investigate this further, HepG2 cells were transfected with either GLUT2 or GLUT8 and with or without TM4SF5 silencing RNA. The cells were starved of glucose for 16 h and then given 450 mg/dL fructose before being lysed for western blot analysis. It was found that the cells with silenced TM4SF5 overexpressed GLUT8 but not GLUT2. Moreover, the presence of lipogenic enzymes that are dependent on TM4SF5 was visibly reduced when GLUT8 was silenced. Those findings suggest that the steatosis pathways that are triggered by high fructose intake are reliant on GLUT8 as the primary fructose transporter in hepatic cells. Targeting GLUT8 or TM4SF5 are promising approaches that have the potential to block the development of non-alcoholic fatty liver disease [95].

### 3.7. Osteonecrosis

Steroid-induced osteonecrosis of the femoral head (SONFH) is a chronic and crippling bone disease which is not yet fully understood despite being fairly common, with nearly 15,000 new reported cases annually in the United States alone. The diagnostic criteria of this disease are fairly well established. However, due to the lack of specific symptoms or biomarkers at the early stages, the majority of patients are diagnosed at the advanced stages, where they will often require total hip replacement surgery. In order to identify new biomarkers that could help in earlier detection of this disease, the authors screened the GSE123568 dataset for differentially expressed genes and identified the module that had the strongest correlation with SONFH (*p* = 7.1 × 10^−278^). Further analysis narrowed that module down to four genes, RHAG, RNF14, HEMGN and SLC2A. Those genes had lower expression in the SONFH group by 33%, 21%, 40% and 30%, respectively. Finally, the ROC curves for those genes showed that all of them were above 0.9, indicating that they could serve as potential diagnostic biomarkers for SONFH.

RHAG is a member of the transporter family SLC42 and is involved in ammonia transport. RNF14 is a prostate coactivator that is involved in the androgen receptor signaling pathway. There have been no studies indicating that those proteins are involved in the development and progression of SONFH. On the other hand, HEMGN is expressed and bone marrow and osteoblasts and is involved in osteoblast recruitment, which means it is likely to be involved in the development of the disease. Finally, SLC2A1 is the sugar transporter GLUT1, and it has been shown in the past that blocking it results in impaired osteoblast anabolic function, differentiation, and mineralization in vitro. The authors concluded that HEMGN and SLC2A1 are the most likely to serve as useful early diagnostic biomarkers of SONFH, and additional experimental validation is needed [96].

### 3.8. Type 2 Diabetes

Trilobatin, a natural food additive found in *Lithocarpus polystachyus* leaves, was shown to significantly reduce high fasting blood glucose levels and insulin resistance in diabetic mice. Examining the mechanism of action revealed that trilobatin activated the Nrf2/ARE signaling pathway that reduces oxidative stress and increased GLUT2 expression by an average of 20% and 25% in the pancreas and liver, respectively, which lowered blood glucose levels and improved insulin sensitivity [97]. A similar effect was observed with the known antihyperglycemic molecule cyanidin-3-O-glucoside, which works on GLUT1 instead of GLUT2 [98].

Another study using a zebrafish model found that 1,25-dihydroxyvitamin D_3_, the most potent vitamin D metabolite, increased the expression of GLUT2 and the insulin receptor *insra*. Those effects were observed only at a high glucose concentration of 20 mM, where the GLUT2 expression was observed to have nearly doubled compared to the control. By contrast, the low glucose concentration of 10 mM resulted in a very small increase in GLUT2 expression that was within the margin of error. In addition, treating diabetic zebrafish with that vitamin D_3_ caused a significant reduction in visible hyperglycemia symptoms, which are growth retardation and the body being curved rather than straight [99].

## 4. Summary and Conclusions

This review contains examples of GLUT involvement in a diverse set of disorders and diseases from the recent literature. Most notably, the involvement of class III GLUTs in those disorders, highlighted in Table 3, warrants further investigation into their structures and roles, as currently the focus in research and available data mostly only focus on class I and GLUT5 of class II. 

In most of the diseases mentioned in this review, especially the cancers, GLUT overexpression was a major underlying factor, and GLUT inhibition or knockdown seemed to help alleviate the disorder. A wide variety of inhibitors with different mechanisms of action and target GLUTs were mentioned in this review, as shown in Table 4. The development of GLUT inhibitors is an active ongoing field of research and has been covered extensively in other reviews [1]. The three examples of GLUT inhibitors included in this review show the two main ways of their development, in-silico screening [18] and natural product screening [14,15]. The overexpressed GLUT transporters in tissues where they are normally not expressed, such as GLUT3 in breast cancer [3], demonstrates their potential as diagnostic biomarkers.

On the other hand, other disorders like osteonecrosis appear to be caused by lower GLUT expression. Using those underexpressed GLUTs as diagnostic biomarkers was also proposed, and treatments might involve managing the diet or using GLUT activators, which seems to be a less popular area of research compared to GLUT inhibitors. There are relatively fewer examples of GLUT activators and their therapeutic potential in the literature, and this might be a topic for a future review.

## Figures and Tables

**Table 1:** Classification, expression, and substrate preference of the 14 known GLUTs.

	Expression Tissues	Main Substrates	
GLUT1	Erythrocytes, blood-tissue barriers	Glucose, 2-DG	Class I
GLUT2	Liver, pancreas, small intestine	Glucose, Glucosamine	
GLUT3	Neurons	Glucose, 2-DG	
GLUT4	Adipocytes, muscle, heart	Glucose, Glucosamine	
GLUT14	Testis	Unknown	
GLUT5	Testis, intestine, muscle	Fructose	Class II
GLUT7	Testis, intestine, prostate	Fructose, glucose	
GLUT9	Liver, kidney	Urate	
GLUT11	Pancreas, kidney, placenta, muscle	Fructose, glucose	
GLUT6	Brain, spleen, leukocytes	Glucose	Class III
GLUT8	Testis, neurons, adipocytes	Glucose, trehalose	
GLUT10	Liver, pancreas	2-DG	
GLUT12	Heart, prostate	Glucose	

**Table 2:** APOE4 associated odds ratios of developing MCI and dementia for different genotypes of transporter genes.

Gene Symbol	SNP ID	Genotype Group	APOE4 Odds Ratio	95% CI	*p* Value
SLC2A1	rs710218	TT	2.35	1.05–5.23	0.037
(GLUT1)		TA + AA	1.3	0.62–2.75	0.49
	rs841851	AA	3.2	1.58–6.46	0.0012
		AG + GG	0.67	0.27–1.67	0.39
SLC24A2	rs1279683	GG	1.21	0.44–3.37	0.71
(SVCT)		GA + AA	2.02	1.05–3.87	0.035

**Table 3:** Summary of diseases and GLUTs in this review.

	Disorder Involved	
GLUT1	Cognitive decline, Epilepsy, Gastric cancer, osteonecrosis	Class I
GLUT2	Type-2 diabetes,	
GLUT3	Breast cancer	
GLUT4	Gastric cancer	
GLUT5	Colon cancer, Chron’s disease	Class II
GLUT6	Endometrial cancer	Class III
GLUT8	Hepatic steatosis	
GLUT10	Arterial tortuosity syndrome, Leukemia	
GLUT12	Breast cancer	

**Table 4:** GLUT inhibitors mentioned in this review.

Inhibitor	Type	Target GLUTs	Associated Disease
Let-7a-5p	miRNA	GLUT12	breast cancer
MSNBA	small molecule	GLUT5	colon cancer
GRg3	natural product	GLUT1, GLUT4	gastric cancer
1:1 CC-DG mixture	natural product	GLUT1	gastric cancer
H22954	lncRNA	GLUT10	leukemia
KL-11743	small molecule	GLUT1-4	TCA cycle deficient tumors
